# Research on Energy Localization and Vibration Suppression of Axially Functionally Graded Porous Beams

**DOI:** 10.3390/ma18184306

**Published:** 2025-09-14

**Authors:** Qiuhua Wang, Rongjiang Tang, Sai Zhang, Kefang Cai, Wenwen Wang, Xuekang Zhang

**Affiliations:** 1 School of Mechanical and Electrical Engineering, Guilin University of Electronic Technology, Guilin 541004, China; wangqiuhua@mails.guet.edu.cn (Q.W.); caikf2015@guet.edu.cn (K.C.); 18486137204@163.com (W.W.); 15954303302@163.com (X.Z.); 2 China Automotive Technology and Research Center Co., Ltd., Tianjin 300300, China; zhangsai@catarc.ac.cn

**Keywords:** functionally graded porous beams, energy localization, vibration control, vibration damping mechanism

## Abstract

Functionally graded porous beam (FGPB) structures are widely used in engineering due to their light weight, high strength, and vibration-damping performance. However, their energy localization and vibration suppression characteristics remain largely unexplored. To address this gap, this study proposes an axially functionally graded porous beam (AFGPB) structure capable of achieving energy localization and suppressing vibration transmission. A semi-analytical model is first developed within the Rayleigh–Ritz framework, using Gaussian functions as basis functions to accurately represent the displacement field. The accuracy of the model is validated by comparing its vibration characteristics with those obtained using the finite element method (FEM). Subsequently, the vibration behavior of double-AFGPB with simply supported boundary constraints is investigated. A series of numerical results are presented in this study to analyze the influence of porosity parameters on the energy localization effect and vibration suppression performance. Results reveal that the porosity power-law index *N* and truncation coefficient *δ* play key roles in energy localization and vibration suppression performance. When *N* ≥ 4, the energy localization effect and the vibration attenuation of the double-AFGPB become more pronounced with increasing *N* and decreasing *δ*, particularly in the low-frequency range.

## 1. Introduction

Functionally graded porous materials (FGPMs) have emerged as core materials in aerospace, automotive, and electronic systems due to their unique structural designs and tunable physical properties [1,2,3,4,5]. By altering the porosity distribution, the mechanical performance of porous structures can be significantly adjusted. Compared to conventional homogeneous structures, FGPMs offer considerable advantages in vibration and noise reduction [5]. The fundamental cause of structural vibration and subsequent noise radiation is the propagation of elastic waves within the structure, along with the interaction between these waves and surrounding media (such as air or water). Therefore, effectively concentrating and dissipating the energy of these waves as they propagate through beams and plates is a promising approach for controlling structural vibration and noise. Consequently, the development of functionally graded porous beam structures capable of enabling wave energy localization and dissipation is of great significance for structural vibration suppression and isolation.

Various theoretical and numerical methods have been applied to investigate the vibration behavior of FGPB. Zhao et al. [6], based on strain gradient theory, developed a size-dependent axial functionally graded (AFG) piezoelectric Euler–Bernoulli nanobeam model, revealing how gradient indices, porosity volume fraction, and distribution patterns affect static bending and free vibration behavior. Reddy et al. [7], using Hamilton’s principle and Reddy’s higher-order beam theory, derived vibration equations to analyze the transverse and axial-free vibration behavior of porous beams under general boundary conditions. Yee et al. [8] formulated coupled dynamic equations for axially graded viscoelastic beams using third-order shear deformation theory, and studied the influence of geometric and material imperfections on natural frequencies and mode shapes. Chen et al. [9] reported that the vibration response of double-beam systems under moving loads can be controlled by tailoring the distribution of bidirectional functionally graded materials. Mutlak et al. [10], employing a nonlocal strain gradient Rayleigh beam model with a power-law porosity distribution, analyzed both forced and free vibration responses using eigenvalue analysis and dynamic amplification factors. Their results demonstrate that optimizing the porosity distribution effectively suppresses vibration amplitudes and enhances structural stability. Several studies [11,12,13] have also noted that increased porosity generally leads to reduced stiffness and lower natural frequencies, while viscoelastic effects help attenuate vibrations. Ebrahimi [14], using Timoshenko beam theory and incorporating shear deformation and rotary inertia, applied a modified power-law model for material gradation in porous Functionally Graded Material (FGM) beams. By combining the Galerkin method and multiscale analysis, the coupling mechanism between porosity and nonlinear vibration was revealed, offering insights for lightweight structural design with improved dynamic stability. Li et al. [15], based on first-order shear deformation theory and isogeometric analysis, conducted a comprehensive study on the static bending, free vibration, and buckling of bidirectional Functionally Graded Porous (FGP) plates, considering the effects of aspect ratio, boundary conditions, porosity distribution, and coefficients. Similar investigations were performed using generalized shear deformation theory and isogeometric methods to analyze FGP plate behavior [16,17]. Chen et al. [18] studied the nonlinear free vibration of shear-deformable sandwich porous beams within the Timoshenko beam framework, examining the effects of porosity coefficient, slenderness ratio, and thickness ratio using detailed numerical simulations. The results offer practical strategies for improving the vibration performance of sandwich porous beams. Qin et al. [19] introduced the Jacobi–Ritz method to analyze both the free and forced vibration of functionally graded porous straight beams under arbitrary boundary conditions. In another contribution, [20] combined third-order shear deformation theory (TSDT) with finite element methods to study the effects of gradient indices and porosity distribution on the vibration behavior of bidirectional functionally graded porous sandwich shells.

To date, no studies have been reported on the use of FGP structures to achieve energy localization. One promising strategy for wave energy localization and dissipation is the Acoustic Black Hole (ABH) concept, which has demonstrated excellent performance in vibration and noise reduction in beam and plate structures [21,22,23,24,25]. The ABH effect is typically achieved by reducing the thickness of a beam or plate according to a power-law function ($h\left(x\right)\;=\;\varepsilon x^{m},m\;\geq \;2$). This gradual reduction causes a continuous variation in structural impedance, leading to a decrease in bending wave velocity, a shortening of the wavelength, and an amplification of the wave amplitude. As a result, wave energy becomes concentrated near the ABH tip, effectively suppressing vibration transmission and enabling energy manipulation and localization [26,27,28,29,30].

However, the ABH structure suffers from inevitable truncation effects due to manufacturing limitations [21,31,32,33]. Researchers have attempted to mitigate wave reflections caused by truncation by adding damping layers or designing cut-off platforms [34,35,36,37,38]. Despite these efforts, the continuously tapered geometry of ABH structures presents challenges in terms of structural safety, sealing, and practical integration. In general, energy localization in structures can be achieved through two mechanisms: (1) thickness variation based on a power-law profile, and (2) spatially varying material properties. Functionally graded materials (FGMs), including porous variants, offer the ability to achieve continuous gradients in both mechanical properties and microstructure. Since both ABH and FGP structures rely on spatial gradients to manipulate wave propagation, it is theoretically feasible to realize energy localization in FGP systems.

Motivated by this insight, the present study proposes a novel AFGPB structure, where the material properties vary continuously along the beam length. A systematic analysis is thereby conducted to investigate the energy concentration effect and vibration control of such AFGPBs. The structure of this paper is organized as follows: Section 2 introduces the theoretical model of the AFGPB and derives the governing equations based on energy principles using Gaussian basis functions to represent the displacement field. The proposed model is validated against finite element simulations. Section 3 investigates and compares the vibration behavior of both double-AFGPB and homogeneous beams (HB). The influence of porosity parameters on energy localization effect and vibration suppression performance is analyzed in the 0~5000 Hz frequency range. Section 4 concludes the paper.

## 2. Mathematical Model of AFGPB

### 2.1. Model Description

**Figure 1:**
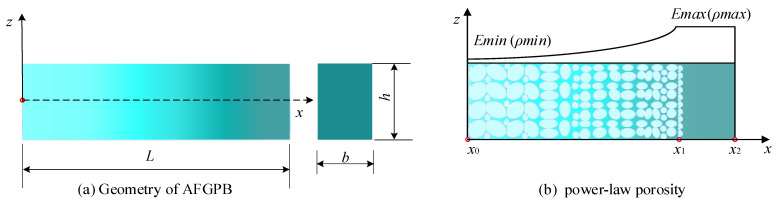
AFGPB geometric model and porosity distributions.

**Figure 2:**
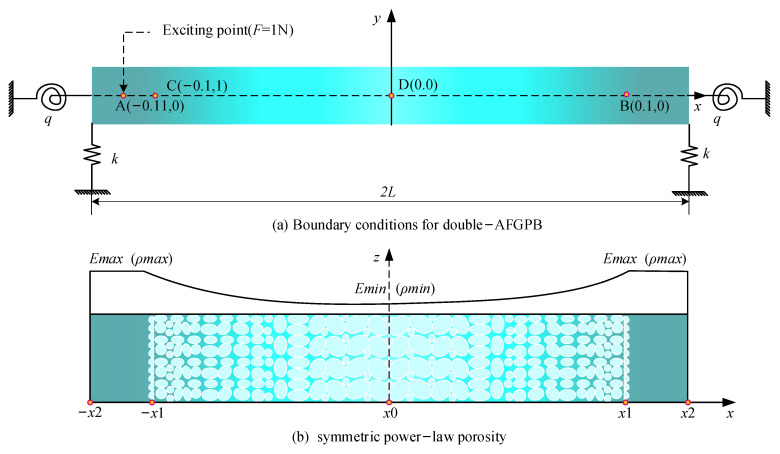
Double-AFGPB geometric mode (**a**) boundary condition; (**b**) porosity distributions.

Figure 1 illustrates the geometric model of the AFGPB with a rectangular cross-section, along with the corresponding porosity distribution. The beam’s length *L*, width *b*, and height *h* are defined along the *x*, *y*, and *z* axes, respectively. The beam consists of two segments: a homogeneous material section (*x*_1_, *x*_2_) and a FGPMs section (*x*_0_, *x*_1_). In the homogeneous section, the porosity is zero. In contrast, in the gradient porous region, the porosity varies along the axial direction following a power-law distribution, with a maximum value less than 1. Figure 2 shows the boundary conditions and porosity distribution of the double-AFGPB, which is symmetrically constructed based on the AFGPB presented in Figure 1. The total length of the beam is 2 *L*, with a width of *b* and a height of *h*. The boundary conditions are simulated using a translational spring *k* and a rotational spring *q*. By adjusting the values of *k* and *q*, different types of boundary conditions can be modeled. For example, *k* = *q* = 0 represent a free boundary, while *k* = ∞ and *q* = 0 correspond to a simply supported boundary.

In this study, the axial porosity *P* distribution is assumed to follow a piecewise function, as defined in Equation (1). In this equation, the truncation coefficient *δ* defines the maximum porosity by controlling the effective length of the graded region, while the porosity power-law index *N* characterizes the degree of material inhomogeneity along the beam axis. Together, *δ* and *N* determine the spatial distribution of density and stiffness, thereby governing the propagation of energy. Their variation is of critical importance for investigating energy localization and vibration suppression in AFGPB.

(1) $$P=\left\{\begin{array}{l} 1-{\left((x+\delta )/L\right)}^{N}(x_{0}\leq x\leq x_{1})\\ 0(x_{1}\leq x\leq x_{2}) \end{array}\right.$$

**Figure 3:**
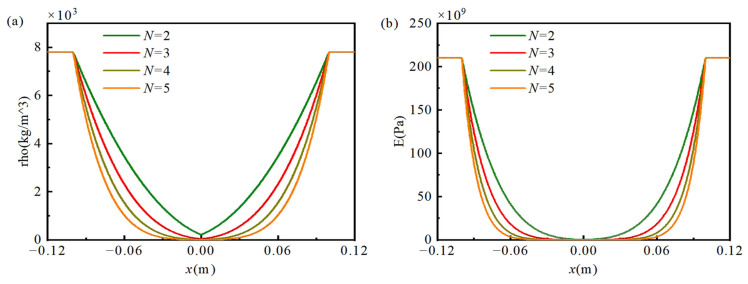
Variation in density and Young’s modulus along the beam length for porosity power-law indices *N* = 2, 3, 4, and 5; (**a**) Density; (**b**) Young’s modulus.

The material properties of the AFGPB can be described using the porosity distribution function in Equation (2), which expresses the beam’s main physical parameters, density and Young’s modulus, as functions of the coordinate *x* and the porosity *P*. In Equation (2), $E_{Uni}$ and $\rho_{Uni}$ represent the Young’s modulus and density of the HB section, and $P\left(x\right)$ represents the porosity function, describing the axial distribution of porosity. As the porosity power-law index *N* varies, the density and Young’s modulus of the AFGPB increase along the axial direction following a power-law distribution. For the double-AFGPB, the material property parameters exhibit a symmetric distribution, as illustrated in Figure 3. Specifically, both the density and Young’s modulus show a large–small–large variation pattern along the beam axis.

(2) $$\begin{array}{l} E(x)=E_{Uni}{\left(1-P(x)\right)}^{2}\\ \rho (x)=\rho_{Uni}(1-P(x)) \end{array}$$

Based on the energy localization effect in the ABH, the accumulated phase of the bending wave propagating from any point on the beam to the beam’s end can be expressed as in Equation (3) [28,39].

(3) $$\Phi (x)=\int_{0}^{x}k(x)dx$$

From the Euler–Bernoulli beam wave number expression, the wave number for the AFGPB is given by Equation (4), where *ω* is the angular frequency, *c* is the phase velocity of the wave, *E*(*x*) is the Young’s modulus of the beam, *ρ*(*x*) is the material density of the beam, *A* is the beam’s cross-sectional area, and *I* is the area moment of inertia.

(4) $$k(x)=\omega /c=\sqrt[{4}]{\rho (x)A\omega^{2}/E(x)I}$$

By substituting Equations (1), (2), and (4) into Equation (3), the accumulated phase integral expression from any point *x* on the beam to the edge of the gradient porous beam structure is obtained.

(5) $$\Phi =\sqrt[{4}]{\frac{12\omega^{2}\rho_{Uni}L^{N}}{h^{2}E_{Uni}}}\int_{0}^{x}{\left(x+\delta \right)}^{\frac{-N}{4}}dx$$

From Equation (5), it can be observed that, when *N* ≥ 4, the accumulated phase Φ tends to infinity. This indicates that bending waves cannot reach the boundary of the graded porous structure, thereby eliminating wave reflection at that edge. As a result, the wave energy is confined and concentrated near the boundary, effectively realizing the energy localization effect.

### 2.2. Derivation of Control Equations

In this study, the shear deformation of the beam and the effect of the moment of inertia about the neutral axis of the cross-section are neglected. Based on the Euler–Bernoulli beam theory [6], the displacement field of the functionally graded porous beam can be represented by Equations (6) and (7):

(6) $$U_{x}(x,z,t)=-z\frac{\partial w_{0}(x,t)}{\partial x}$$

(7) $$W_{z}(x,z,t)=w_{0}(x,t)$$

where $w_{0}$ represents the displacement in the *z*-direction, with *t* denoting time. The geometric, physical, and equilibrium equations for the AFGPB are given by Equations (8)–(10):

(8) $$\varepsilon_{x}=\frac{\partial U_{x}}{\partial x}=-z\frac{\partial^{2}w}{\partial x^{2}}$$

(9) $$\sigma_{x}=E(x)\varepsilon_{x}=-E_{U\mathrm{ni}}{\left(1-p(x)\right)}^{2}\cdot z\frac{\partial^{2}w_{0}}{\partial x^{2}}$$

(10) dM/dx=Q,dQ/dx=q

In Equation (10), *M* and *Q* represent the bending moment and shear force at the beam’s cross-section, respectively, while q represents the uniformly distributed transverse load acting on the beam. By integrating the normal stress over the cross-sectional area, the expression for the bending moment is given by Equation (11), where $I=\int_{A}Z^{2}dA$ is the area moment of inertia.

(11) $$M=b\int_{\frac{-h}{2}}^{\frac{h}{2}}\sigma_{x}zdz=-b\int_{\frac{-h}{2}}^{\frac{h}{2}}E(x)z^{2}\frac{\partial^{2}w_{0}}{\partial x^{2}}dz=-E(x)I\frac{\partial^{2}w_{0}}{\partial x^{2}}$$

Considering the effects of axial deformation, variable density, and modulus, the kinetic energy ***T*** and potential energy ***U*** of the AFGPB are given by Equations (12) and (13), respectively.

(12) $$T=\frac{1}{2}\int_{x_{0}}^{x_{2}}\rho (x)A{\left(\frac{\partial w}{\partial t}\right)}^{2}dx$$

(13) $$U=\frac{1}{2}\int_{0}^{L}E(x)I{\left(\frac{\partial^{2}w}{\partial x^{2}}\right)}^{2}dx$$

After determining the system’s energy, the Hamiltonian variational principle is applied. In the interval [0, *t*], for all possible system states, the true motion of the conservative system minimizes the functional. This results in the first-order variation in the Hamiltonian action being zero, which leads to the following derivation of the vibrational differential equation for the AFGPB:

(14) $$L=\int_{0}^{t}\left(T-U\right)dt$$

By applying the extremum condition, Equations (12) and (13) are substituted into Equation (14).

(15) $$\delta L=\int_{0}^{t}\int_{0}^{L}\left[\rho (x)A\frac{\partial w}{\partial t}\delta \left(\frac{\partial w}{\partial t}\right)-E(x)I\frac{\partial^{2}w}{\partial x^{2}}\delta \left(\frac{\partial^{2}w}{\partial x^{2}}\right)\right]dxdt=0$$

Using the method of distributed integration, the final free vibration control equation for the AFGPB is derived as follows:

(16) $$\rho_{Uni}(1-P(x))A\frac{\partial^{2}w}{\partial t^{2}}+E_{Uni}{\left(1-P(x)\right)}^{2}I\frac{\partial^{4}w}{\partial x^{4}}=0$$

### 2.3. AFGPB Semi-Analytical Model

Due to the continuous gradient variation in porosity within the AFGPB structure, there is an issue of non-uniform distribution of the bending wave’s wavelength along the beam. Direct analytical methods for modeling and solving such a system would require significant computational effort, and, in some cases, may not even be solvable. The semi-analytical method combines the accuracy of analytical approaches with the flexibility of numerical methods in handling boundary conditions. This method has been widely used in the theoretical modeling of beam and plate structures.

Therefore, in this study, a semi-analytical model is established for the AFGPB, and its accuracy is validated using the finite element method. The key to the semi-analytical approach lies in selecting an appropriate set of basis functions to describe the bending vibration field of the beam. The Gaussian function is chosen as the basis function due to its high differentiability and the absence of singularities, making it particularly suitable for describing the rapidly varying displacement field in the gradient porous region [38,40]. The form of the Gaussian basis function is as follows [38,41]:

(17) $$\varphi_{i}(x)=2^{\frac{j}{2}}\exp\left[-\frac{{\left(2^{j}x-p\right)}^{2}}{2}\right]$$

where j represents the scale factor, which describes the extent of stretching or compression of the Gaussian function, and p is the translation factor.

As discussed in [38,41], the effective interval of the basis function cannot exceed the total length of the beam, and the lower bound of the scale factor is determined as follows:

(18) $$j\geq \mathrm{ceil}\left[\log_{2}\left(8/L\right)\right]$$

where ceil (※) represents the ceiling function.

Additionally, the translation factor p indicates the center of the Gaussian function, and the translated basis function’s effective interval must intersect with the beam’s domain to avoid matrix singularity. Hence, the valid set for the translation factor p is defined as follows [38,41]:

(19) $$p=\left[-4+\mathrm{floor}(-2^{j}x_{0}),\;\mathrm{ceil}(2^{j}x_{1})+4\right]$$

where floor (※) represents the floor function.

The deflection of the AFGP beam structure is defined as a combination of the basis functions and a set of unknown weight coefficients [38,41]:

(20) $$w(x,t)=\sum_{i}^{n}a_{i}(t)\varphi_{i}(x)=a^{T}\varphi =\varphi^{T}a$$

where ***φ*** is the vector of basis functions and ***a*** is the set of weight coefficients.

Using the Rayleigh–Ritz method, a semi-analytical model for the free vibration of the AFGPB structure is established with Gaussian functions as the basis. The first step is to derive the system’s kinetic energy *T*, potential energy *U*, and work performed by external forces. By substituting the deflection function into the energy expressions Equations (12) and (13), the following final expressions are obtained [38]:

(21) $$\begin{array}{l} T=\frac{1}{2}\int_{x_{0}}^{x_{2}}\rho (x)A{\left(\sum_{i=1}^{n}{\dot{a}}_{i}\varphi_{i}\right)}^{2}dx\\ =\frac{1}{2}{\dot{a}}^{T}\left[\int_{x_{0}}^{x_{2}}\rho (x)A\varphi \varphi^{T}dx\right]\dot{a}\\ =\frac{1}{2}{\dot{a}}^{T}M\dot{a} \end{array}$$

In Equation (21), **M** represents the mass matrix.

(22) $$M=\int_{x_{0}}^{x_{2}}\rho (x)A\varphi \varphi^{T}dx=\int_{x_{0}}^{x_{1}}\rho_{AFGP}(x)A\varphi \varphi^{T}dx+\int_{x_{1}}^{x_{2}}\rho_{U\mathrm{ni}}A\varphi \varphi^{T}dx$$

Similarly, the potential energy expression is given by Equation (23) [38]:

(23) $$\begin{array}{l} U=\frac{1}{2}{\int_{x_{0}}^{x_{2}}E(x)I\left(\sum_{i=1}^{n}a_{i}{\varphi_{i}}^{\prime \prime }\right)}^{2}dx\\ =\frac{1}{2}a^{T}\left[\int_{x_{0}}^{x_{2}}E(x)I\varphi^{\prime \prime }{\varphi \prime \prime }^{T}dx\right]a\\ =\frac{1}{2}a^{T}Ka \end{array}$$

where **K** denotes the stiffness matrix:

(24) $$K=\int_{x_{0}}^{x_{2}}E(x)I\varphi^{\prime \prime }{\varphi \prime \prime }^{T}dx=\int_{x_{0}}^{x_{1}}E_{AFGP}(x)I\varphi^{\prime \prime }{\varphi \prime \prime }^{T}dx+\int_{x_{1}}^{x_{2}}E_{Uni}I\varphi^{\prime \prime }{\varphi^{\prime \prime }}^{T}dx$$

The work performed by external forces is expressed as follows:

(25) $$W=\int_{x_{0}}^{x_{2}}f(t)w(x,t)\mathrm{dx}=a^{T}\int_{x_{0}}^{x_{2}}f(t)\varphi \mathrm{dx}=a^{T}f$$

where **f** is the external force vector in generalized coordinates.

By combining Equations (21), (23), and (25), and using the Euler–Lagrange equation, the equation of motion is derived in the form of Equation (26) [38].

(26) $$Ma^{\prime \prime }(t)+Ka(t)=f(t)$$

Let the external force $f(t)=Fe^{i\omega t}$ represent a steady-state harmonic excitation and the response vector $a(t)=ae^{i\omega t}$ represent the system’s dynamic response. Substituting into Equation (26) and eliminating the time-harmonic terms, the system’s dynamic equation in the frequency domain is obtained as follows [38]:

(27) $$\left(K-\omega^{2}M\right)A=F$$

For the beam’s free vibration analysis, setting F = 0 allows for the calculation of the characteristic frequencies and eigenvectors of the AFGPB. A dynamic model for the double-AFGPB can be established using the same approach. Since the double-AFGPB is constructed symmetrically from a single AFGPB, its theoretical derivation is omitted here for brevity.

## 3. Numerical Results

In this section, the accuracy and validity of the semi-analytical model are verified from two perspectives, free-vibration modes and forced-vibration responses, using finite element (FE) results as a reference. On this basis, a comprehensive analysis is conducted to investigate the effects of porosity parameters, including the power-law index *N* and truncation coefficient *δ*, on the vibration characteristics of the double-AFGPB.

### 3.1. Model Validation

#### 3.1.1. Free Vibration Mode Validation

For the numerical validation, an AFGPB made of steel with a length *L* = 0.12 m—where *x*_0_ = 0 m, *x*_1_ = 0.1m, and *x*_2_ = 0.12m—a width *b* = 0.001 m, and a height *h* = 0.003 m was considered, as illustrated in Figure 1. Its physical properties are as follows [38]: $\rho_{Uni}=7800\;kg/m^{3}$, $E_{Uni}=210$ Gpa, ν= 0.3. The porosity truncation coefficient was set to *N* = 4, *δ* = 0.02. In the numerical validation, the Euler–Bernoulli beam model in COMSOL Multiphysics 6.3 was employed to construct the AFGPB. The one-dimensional beam was discretized using edge elements, and a mesh independence study was performed to ensure the reliability of the simulation results. Five mesh schemes were considered, with the number of elements ranging from coarse to fine being 240, 480, 960, 1440, and 1920 respectively. The 10th, 20th, 30th, and 40th eigen-frequencies of the beam were calculated for each mesh configuration. The variation in natural frequencies with increasing mesh densities was then analyzed to verify mesh convergence. For the convenience of analysis, the relative error is defined as $\varepsilon_{i}=\left|Q_{i}-Q_{\mathrm{fine}}\right|/Q_{i}\times 100\%$, where Q_fine_ represents the calculation result of the finest mesh, where i ranges from 1 to 4, representing the other four mesh schemes. The results, summarized in Table 1, show that when the mesh number reaches 480, the relative errors of the four selected natural frequencies are all below 2%. Moreover, as the mesh is further refined, the relative errors gradually decrease. Based on these results, the numerical model is considered to have achieved mesh independence. To balance computational accuracy and efficiency, the beam was discretized into 480 edge elements, resulting in 1443 degrees of freedom, with a mesh size of 0.25 mm. To improve the readability and general applicability of the results, all displacements were expressed in dimensionless form. The dimensionless modal displacement ψ is defined as follows:

(28) $$\psi =\frac{w}{w_{0}}$$

where $w_{0}$ is the modal maximum displacement at position $x_{0}$.

**Figure 4:**
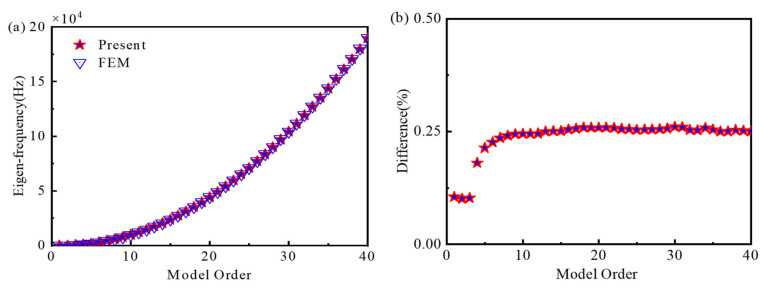
Comparisons of (**a**) eigen-frequencies and (**b**) relative error against the FEM results.

**Figure 5:**
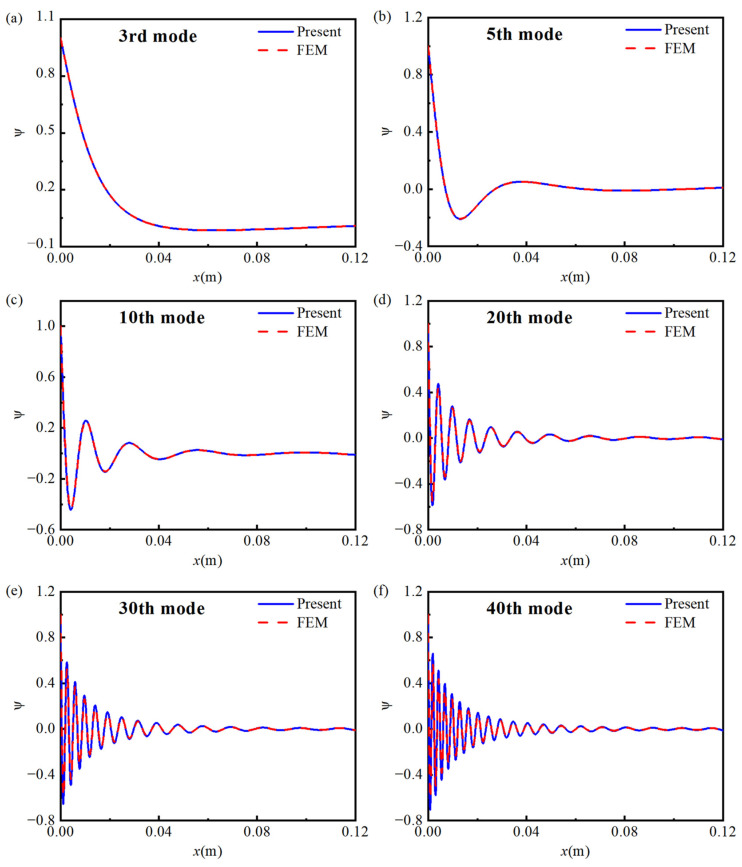
Mode shapes obtained from both the theoretical (semi-analytical) and finite element models for different vibration orders. (**a**) 3rd; (**b**) 5th; (**c**) 10th; (**d**) 20th; (**e**) 30th; (**f**) 40th.

Figure 4a presents a comparison of the first 40 eigen-frequencies obtained from the Gaussian expansion-based semi-analytical model and the finite element model. The relative errors ($\mathrm{Difference}={(\mathrm{freq}}_{\mathrm{FEM}}-{\mathrm{freq}}_{\mathrm{Present}})/{\mathrm{freq}}_{\mathrm{FEM}}\times 100\%$) are shown in Figure 4b. The results indicate good agreement between the two methods, with the relative error of all first 40 eigen-frequencies kept within 1%. Furthermore, Figure 5 illustrates the modal shapes from both methods were compared for the 3rd, 5th, 10th, 20th, 30th, and 40th modes. The results demonstrate that the mode shapes obtained from both methods are nearly identical, further confirming the accuracy and reliability of the semi-analytical model based on the Gaussian expansion method.

It can be observed that, in the uniform section of the beam, the bending wave’s wavelength and amplitude remain largely unchanged. However, in the graded porous section, as the mode order increases, the bending wave’s wavelength is gradually compressed, and the amplitude significantly increases. This leads to the concentration of vibrational energy near the edge of the porous structure, effectively achieving an energy localization effect, which is consistent with the energy localization characteristics of traditional ABH structures. These results confirm that, by properly designing the porosity distribution and inducing a gradient in the density and Young’s modulus, wave energy localization can be effectively realized.

**Figure 6:**
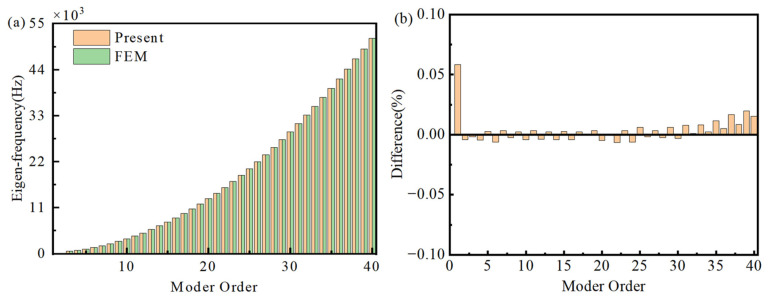
Comparisons of (**a**) eigen-frequencies and (**b**) relative error against the FEM result.

**Figure 7:**
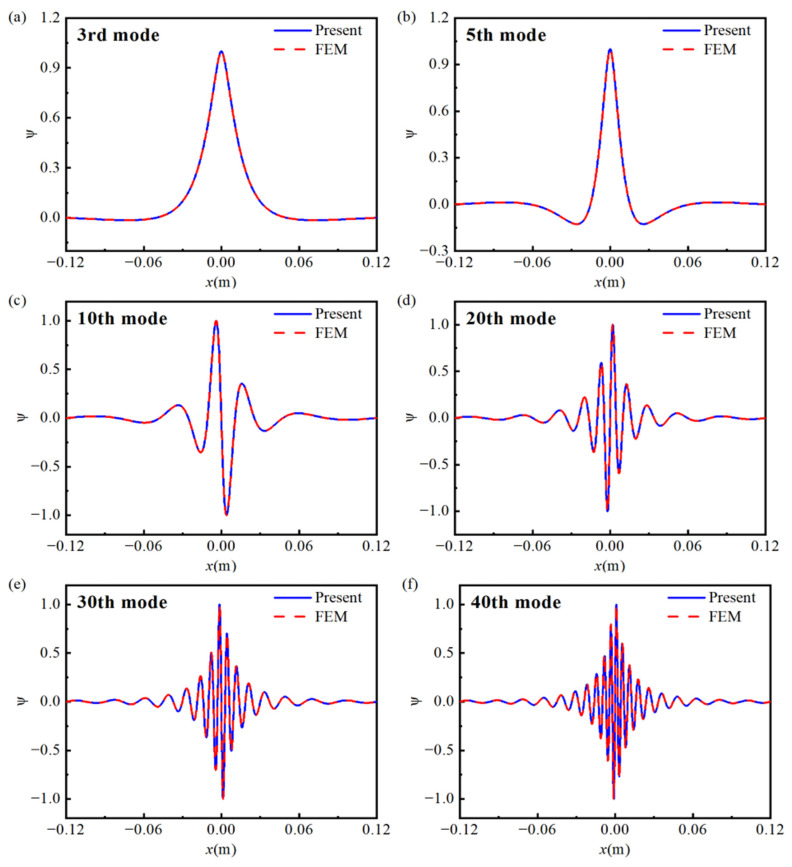
Partial mode shapes of the double-AFGPB: (**a**) 3rd; (**b**) 5th; (**c**) 10th; (**d**) 20th; (**e**) 30th; (**f**) 40th.

Figure 6 presents the first 40 eigen-frequencies of the double-AFGPB with simply supported boundaries. The results show that the deviation between the semi-analytical model and the Finite Element Analysis(FEA) is less than 1%, indicating that the proposed semi-analytical model can accurately predict the vibrational characteristics of the structure. Furthermore, Figure 7 illustrates selected mode shapes of the double-AFGPB. It can be observed that the maximum displacement occurs near the center of the beam, where the porosity is highest. This finding is consistent with the earlier results for the single AFGPB. Therefore, it can be concluded that double-AFGPBs are also capable of achieving energy localization, which lays a theoretical foundation for research on structural vibration suppression.

#### 3.1.2. Forced Response Validation

**Figure 8:**
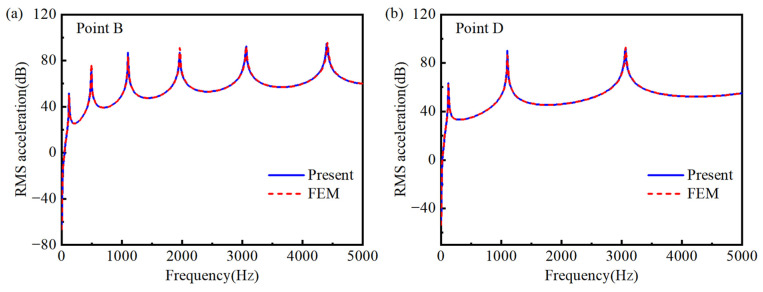
Comparison between the semi-analytical model and the finite element model for the HB conducted at (**a**) point B and (**b**) point D.

**Figure 9:**
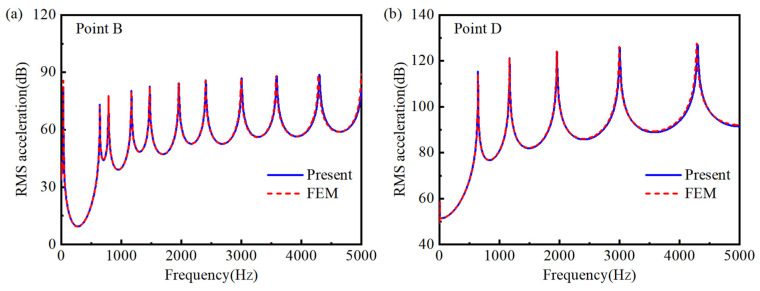
Comparison between the semi-analytical model and the finite element model for the double-AFGPB conducted at (**a**) point B and (**b**) point D.

To further validate the semi-analytical model, the forced vibration response of both the double-AFGPB (Figure 2) and a HB with identical dimensions are analyzed. Both ends of the beam are simply supported. The geometric parameters are defined as follows: *L* = 0.24 m, *b* = 0.001 m, and *h* = 0.003 m. The material properties are as follows: $\rho_{U\mathrm{ni}}$ = 7800 kg/m^3^, $E_{U\mathrm{ni}}$ = 210 GPa, ν= 0.3, loss factor η= 0.003. To ensure consistency in comparison, the porosity parameters for the double-AFGPB are set to *N* = 4 and *δ* = 0.02. Specifically, a harmonic force of 1 N is applied at the excitation point A (−0.11, 0). To ensure date reliability, root mean square (RMS) acceleration values $a_{\mathrm{RMS}}\left(\mathrm{dB}\right)\;=\;20\times \log10\left(\left|a_{w}\right|/\sqrt{2}\right)$ [38] are extracted at the measurement points B (0.1, 0) and D (0, 0). Figure 8 and Figure 9 show the frequency-domain responses at points B and D for the HB and the double-AFGPB, respectively. The results from the two approaches exhibit good agreement, further confirming the accuracy of the proposed semi-analytical model. This validation provides a solid theoretical foundation for the subsequent investigation of the vibration behavior of double-AFGPB.

### 3.2. Parameter Analysis

Building upon the validated model, this section further explores the vibration behavior of the double-AFGPB. Based on modal analysis results and forced response, the effects of key porosity parameters on energy localization, vibration response are systematically analyzed, including the power-law index *N* and truncation coefficient *δ*. This analysis aims to comprehensively evaluate the energy concentration effect and vibration suppression performance of the beam from multiple perspectives.

#### 3.2.1. Influence of Porosity Index *N* and Truncation Coefficient δ on Vibration Energy Localization

Mode shapes describe the structural vibration patterns at eigen-frequencies and reflect the spatial distribution of displacement. As vibration energy is proportional to the square of displacement, regions with higher amplitudes indicate areas of energy localization. Thus, analyzing mode shape distributions provides insight into both the location and intensity of energy localization. This section provides a comprehensive analysis of the effects of porosity parameters on energy localization and examines the sensitivity of the structure’s eigen-frequencies to these parameters.

**Figure 10:**
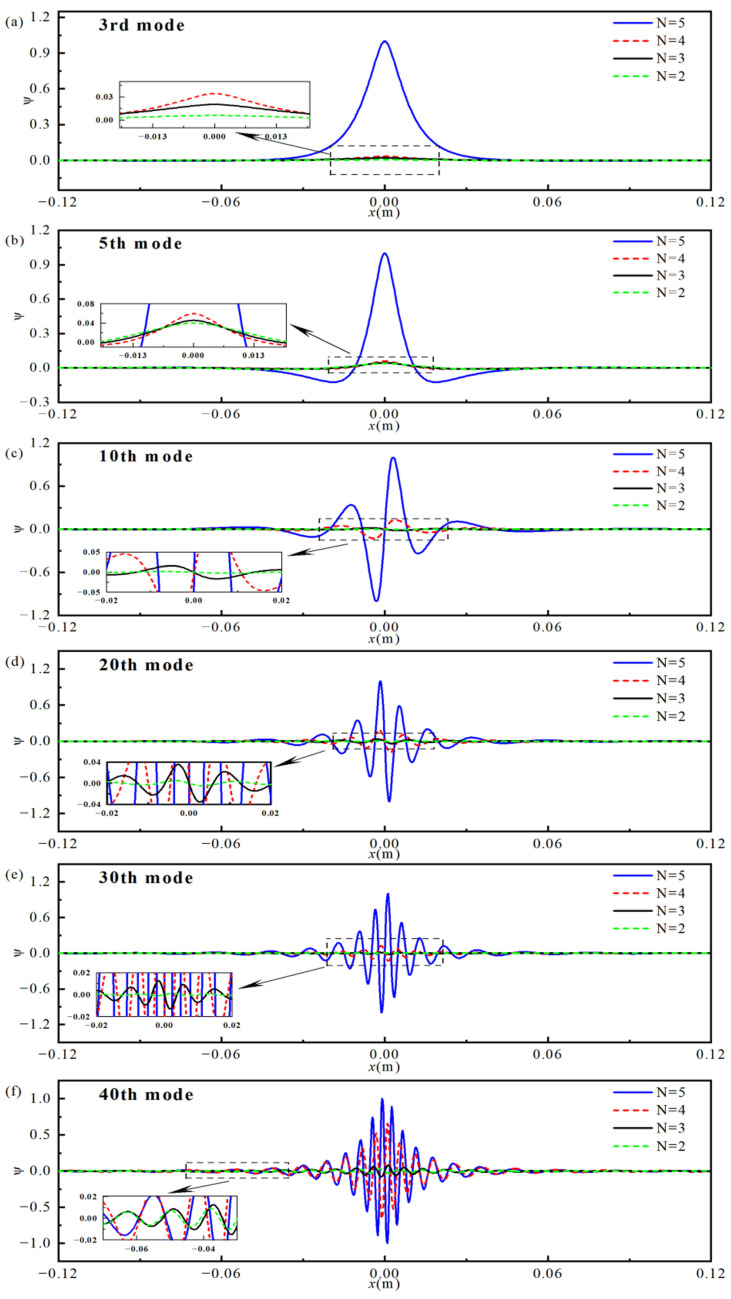
Mode shapes for different porosity power-law indices *N*: (**a**) 3rd; (**b**) 5th; (**c**) 10th; (**d**) 20th; (**e**) 30th; (**f**) 40th.

Figure 10 presents the 3rd, 5th, 10th, 20th, 30th, and 40th mode shapes of the double-AFGPB for different porosity indices (*N* = 2, 3, 4 and 5). The horizontal axis represents the beam’s axial coordinate, while the vertical axis shows the dimensionless modal displacement ψ. As seen in the figure, for a given mode, increasing the porosity index *N* leads to higher displacement amplitudes near the beam’s center. This suggests that larger values of *N* enable smoother impedance transitions, allowing for bending wave energy to concentrate more effectively near the region with maximum porosity. When *N* = 2 or 3, the energy localization effect is weak due to strong wave reflections and scattering caused by impedance mismatch. In contrast, the case of *N* = 5 shows significantly enhanced energy convergence compared to *N* = 4. From the 3rd to the 40th mode, bending waves compress and converge rapidly toward the beam center, exhibiting shorter wavelengths and larger peak amplitudes. These results indicate a stronger energy localization effect and validate the theoretical condition for energy localization described in Equation (5).

**Figure 11:**
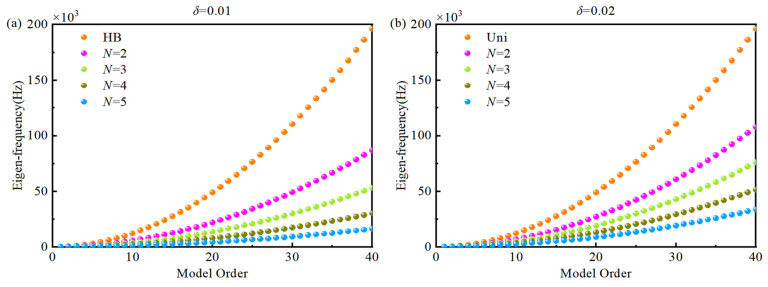
Comparison of the first 40 eigen-frequencies for different porosity indices *N*: (**a**) *δ* = 0.01, (**b**) *δ* = 0.02.

Figure 11 shows the first 40 eigen-frequencies of a simply supported double-AFGPB for different porosity indices (*N* = 2, 3, 4, 5) compared to a HB of the same dimensions. The results indicate that increasing the porosity index *N* leads to a greater reduction in eigen-frequencies. This occurs because a higher *N* more significantly alters the distribution of effective mechanical properties such as density and Young’s modulus. As *N* increases, variations in stiffness and inertia become more pronounced, causing a consistent decrease in the beam’s eigen-frequencies. Furthermore, the reduction in frequency becomes more substantial as *δ* decreases. A smaller *δ* increases the maximum porosity, which in turn lowers the beam’s effective stiffness and stiffness-to-mass ratio, leading to further frequency reduction.

#### 3.2.2. Analysis of Vibration Suppression Effect of Double-AFGPBs Under Different Porosity Indices *N* and Truncation Coefficients *δ*

The previous section has confirmed that variations in porosity parameters simultaneously affect both the energy localization intensity and natural frequencies of double-AFGPBs. Since the magnitude and spatial distribution of material density and Young’s modulus are directly influenced by these parameters, this section further analyzes the effects of the aforementioned parameters on the vibration suppression performance of double-AFGPBs based on steady-state response results.

**Figure 12:**
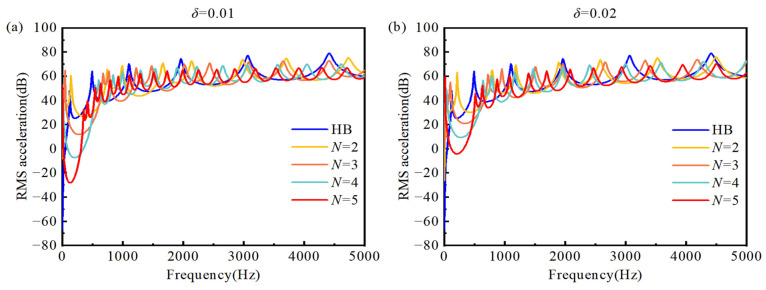
Acceleration frequency responses at measurement point B under different porosity indices *N*: (**a**) *δ* = 0.01; (**b**) *δ* = 0.02.

Figure 12 presents the acceleration frequency responses of the double-AFGPB for different porosity power-law indices *N* and truncation coefficients *δ*. As shown in Figure 12a,b, the resonance peaks of the double-AFGPB are significantly lower than those of the HB at the same measurement locations. As *N* increases, vibration attenuation becomes more evident within the 0~1000 Hz range. Notably, for *N* = 4 and 5, the suppression of vibrations below 500 Hz is more pronounced compared to *N* = 2 and 3. This is mainly because, when *N* ≥ 4, bending waves begin to exhibit energy localization near the beam center, effectively reducing vibration transmission to the right end. Furthermore, the effect of the truncation coefficient *δ* on the frequency response indicates that larger *N* combined with smaller *δ* enhances energy dissipation. For example, when *N* = 5 and *δ* = 0.01, the acceleration amplitude drops to −30 dB, which is nearly a twofold reduction compared to the HB in the 0~500 Hz range. Interestingly, while higher *N* and smaller *δ* improve vibration attenuation, they also narrow the attenuation bandwidth and introduce additional resonance peaks. This is attributed to changes in the effective mass distribution and stiffness matrix due to porosity variations, which increase the number of vibrational modes in the beam.

**Figure 13:**
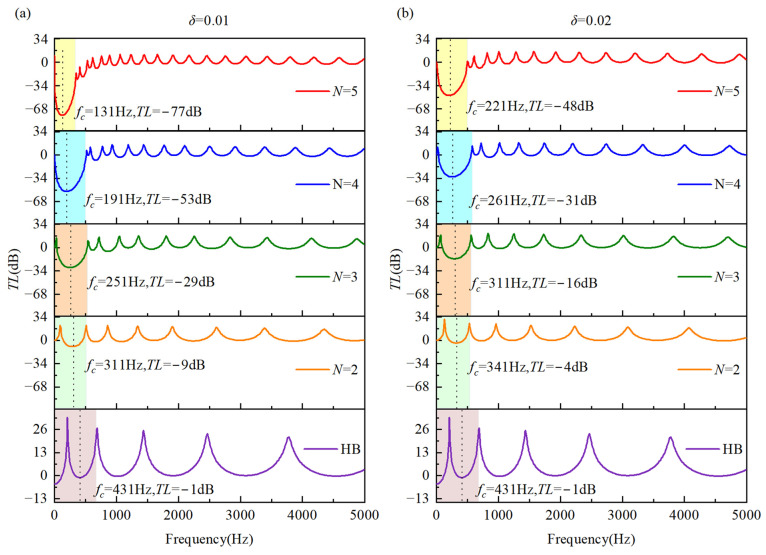
Shows the *TL* curves in the 0~5000 Hz range for different values of porosity index *N*: (**a**) *δ* = 0.01; (**b**) *δ* = 0.02..

**Figure 14:**
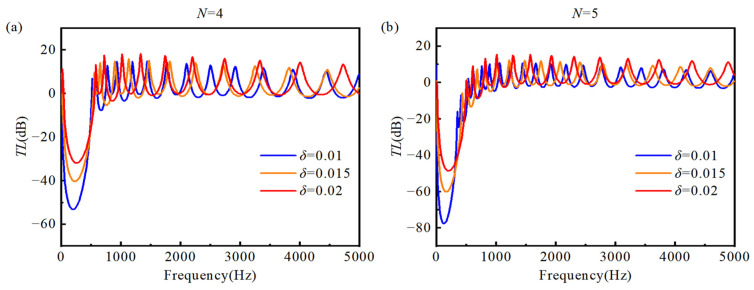
Shows the *TL* curves in the 0~5000 Hz range for different values of truncation coefficient *δ*: (**a**) *N* = 4; (**b**) *N* = 4.

In order to quantify the vibration suppression performance of the double-AFGPB, the transmission loss ($TL=20\log10abs\left(a_{wB}/a_{wA}\right)$) is defined as the ratio of the acceleration amplitude at the right-side response point B to that at the left-side excitation point A. A negative *TL* value indicates that the vibrational energy at the response point is lower than that at the excitation point, reflecting the beam’s capacity for vibration attenuation and isolation under harmonic excitation. Figure 13 presents the *TL* curves over the 0~5000 Hz frequency range for different porosity indices *N*, with truncation coefficients *δ* set to 0.01 and 0.02. The HB exhibits negligible ability to block vibrational energy transmission. In contrast, the AFGPB exhibits excellent vibration suppression performance over the entire frequency range, following a trend similar to that observed in the acceleration frequency response curves. As shown in Figure 13a,b, increasing *N* results in a lower *TL* values, indicating stronger vibration attenuation. Furthermore, as *N* increases, the attenuation bandwidth in the 0~500 Hz range becomes narrower, and the center frequency *f_c_* shifts toward the lower end. Notably, for *N* = 5 and *δ* = 0.01, the *TL* reaches −77 dB at a center frequency *f_c_* of 131 Hz approximately 40% lower than that for *δ* = 0.02. Figure 14 presents the *TL* curves for truncation coefficients *δ* = 0.01, 0.015, and 0.02 at porosity indices *N* = 4 and *N* = 5. It can be observed that even slight variations in *δ* result in a significant reduction in *TL*. Moreover, the double-AFGPB exhibits a stronger vibration suppression capability at *N* = 5 compared to *N* = 4, which is consistent with the previously discussed findings.

In summary, the vibration attenuation and isolation performance of the double-AFGPB improves with increasing *N* and decreasing *δ*. This enhancement is primarily attributed to the greater non-uniformity in equivalent stiffness distribution along the beam at higher *N*, which intensifies impedance mismatches during wave propagation and promotes energy dissipation. These findings demonstrate that the vibration attenuation characteristics of AFGPB in the low-frequency range can be effectively tailored by tuning the porosity index *N* and the truncation coefficient *δ*. Moreover, due to their tunability and design flexibility, such structures offer great potential for lightweight, high-strength solutions in broadband, low-frequency vibration isolation, and damping.

## 4. Conclusions

This study proposes an AFGPB as a novel structural configuration to achieve energy localization and suppress structural vibration. A semi-analytical dynamic model based on the Rayleigh Ritz method and Gaussian basis functions was developed to accurately predict the dynamic response of AFGPB. A series of numerical examples are presented by comparing the results obtained with FEM and the present method, demonstrating excellent agreement in both free and forced vibration response. The model was validated using comprehensive comparisons with finite element simulations, demonstrating excellent agreement in both free and forced vibration scenarios. Parametric studies reveal that both the porosity power-law index *N* and truncation coefficient *δ* significantly influence the beam’s dynamic behavior. Specifically, larger values of *N* and smaller values of *δ* enhance energy localization and vibration attenuation, especially in the 0~500 Hz range. Mode shape analysis confirmed that bending waves are effectively trapped near the center of the beam, and transmission loss results show that the structural vibration suppression capability is enhanced with increasing non-uniformity in stiffness and mass distribution. This demonstrates excellent low-frequency vibration suppression performance and lightweight properties of the double-AFGPB in vibration control. Furthermore, due to the manufacturing challenges associated with the sample preparation process, this study only theoretically investigates the energy localization and vibration suppression performance of AFGPB, and no experimental validation has been conducted so far.

## Figures and Tables

**Table 1:** Mesh convergence study.

Mesh Element Count	10th (Hz)	Relative Error ε (%)	20th (Hz)	Relative Error ε (%)	30th (Hz)	Relative Error ε (%)	40th (Hz)	Relative Error ε (%)
240	9809.3	0	44,320	0.0045	104,390	0.077	199,940	7.11
480	9809.3	0	44,318	0	104,380	0.067	190,000	1.79
960	9809.3	0	44,318	0	104,380	0.067	188,040	0.74
1440	9809.3	0	44,318	0	104,380	0.067	187,020	0.19
1920	9809.3	0	44,318	0	104,310	0	186,660	0

## Data Availability

The original contributions presented in this study are included in the article. Further inquiries can be directed to the corresponding author.
